# Association testing of copy number variants in schizophrenia and autism spectrum disorders

**DOI:** 10.1186/1866-1955-4-15

**Published:** 2012-05-30

**Authors:** Bernard J Crespi, Helen J Crofts

**Affiliations:** 1 Department of Biosciences, Simon Fraser University, Burnaby, BC, V5A 1 S6, Canada

**Keywords:** Autism, Schizophrenia, Copy Number Variants

## Abstract

**Background:**

Autism spectrum disorders and schizophrenia have been associated with an overlapping set of copy number variant loci, but the nature and degree of overlap in copy number variants (deletions compared to duplications) between these two disorders remains unclear.

**Methods:**

We systematically evaluated three lines of evidence: (1) the statistical bases for associations of autism spectrum disorders and schizophrenia with a set of the primary CNVs thus far investigated, from previous studies; (2) data from case series studies on the occurrence of these CNVs in autism spectrum disorders, especially among children, and (3) data on the extent to which the CNVs were associated with intellectual disability and developmental, speech, or language delays. We also conducted new analyses of existing data on these CNVs in autism by pooling data from seven case control studies.

**Results:**

Four of the CNVs considered, dup 1q21.1, dup 15q11-q13, del 16p11.2, and dup 22q11.21, showed clear statistical evidence as autism risk factors, whereas eight CNVs, del 1q21.1, del 3q29, del 15q11.2, del 15q13.3, dup 16p11.2, dup 16p13.1, del 17p12, and del 22q11.21, were strongly statistically supported as risk factors for schizophrenia. Three of the CNVs, dup 1q21.1, dup 16p11.2, and dup 16p13.1, exhibited statistical support as risk factors for both autism and schizophrenia, although for each of these CNVs statistical significance was nominal for tests involving one of the two disorders. For the CNVs that were statistically associated with schizophrenia but were not statistically associated with autism, a notable number of children with the CNVs have been diagnosed with autism or ASD; children with these CNVs also demonstrate a high incidence of intellectual disability and developmental, speech, or language delays.

**Conclusions:**

These findings suggest that although CNV loci notably overlap between autism and schizophrenia, the degree of strongly statistically supported overlap in specific CNVs at these loci remains limited. These analyses also suggest that relatively severe premorbidity to CNV-associated schizophrenia in children may sometimes be diagnosed as autism spectrum disorder.

## Background

Recent studies of schizophrenia and autism spectrum disorders have generated large suites of data, indicating that each of these disorders is mediated in part by rare copy number variants (CNVs), with substantial overlap in copy number risk loci between the two disorders [1-8]. These data are important because they provide novel insights into both the neurodevelopmental causes of autism spectrum disorders and schizophrenia, and the relationship between the two disorders, a long-standing unresolved issue in psychiatry with direct implications for etiology, diagnosis, the design of research programs, and therapy.

Overlap in copy number loci or variants between schizophrenia and autism spectrum disorders may have several possible causes. First, CNV loci may overlap between the two disorders, but the actual variants associated with each of them, such as deletions versus duplications of the same region, or different specific regions, may vary within and between the two disorders. Examples of genes involved in CNVs for this category include APBA2, CNTNAP2, NRXN1, PARK2, and SHANK3 [9-13].

Second, the same CNV may have been reported among individuals with schizophrenia or autism spectrum disorders, or statistically supported as a risk factor for both disorders. Such findings implicate overlapping genetic risk factors and shared etiology, which has been postulated to help explain shared phenotypes, mainly deficits and abnormalities in social development and language. This interpretation, however, runs counter to a large body of non-genetic evidence that differentiates autism spectrum disorders from schizophrenia [14-17], including an exclusionary condition with regard to autism in DSM-IV. How can this apparent incongruity be resolved? One possible explanation is that shared phenotypes of autism spectrum disorders and schizophrenia could be underlain by shared CNV risk factors. This hypothesis has yet to be addressed directly and requires more detailed information on genotype-phenotype relations than is currently available. By contrast, an alternative yet non-exclusive hypothesis, originally suggested by Eliez [18] in the context of 22q11.2 deletions, is that for some loci, sharing of CNVs between schizophrenia and autism is more apparent than real, because of false-positive diagnoses of relatively severe, CNV-associated premorbidity to schizophrenia as autism or autism spectrum disorder [19]. The presence of such false positives is predicated on the supposition that autism or autism spectrum disorders and schizophrenia represent distinct conditions with partially overlapping childhood psychological deficits and abnormalities, which are due to different neurodevelopmental causes.

In this article, data from CNV studies of schizophrenia and autism spectrum disorders are used to evaluate alternative hypotheses [20] for the relationship between these disorders that follow from these considerations, and to evaluate the plausibility of the hypothesis of diagnoses of premorbidity to schizophrenia as an autism spectrum disorder in individuals with CNVs. The primary alternative hypotheses considered are: (1) schizophrenia and autism spectrum disorders as conditions that are genetically distinct with regard to their associations with CNVs; and (2) schizophrenia and autism spectrum disorders as overlapping with regard to their associations with CNVs. Such overlap may, however, either be real (a true positive) or only apparent and due substantially to false positive diagnoses of premorbidity to schizophrenia as an autism spectrum disorder. These hypotheses make alternative, more or less exclusive predictions (Table 1) that are evaluated using data from CNVs in these two disorders, and data on other phenotypes and diagnoses associated with the CNVs.

**Table 1:** Predictions for the distributions of autism or ASD and schizophrenia in children and adults under different models of CNV specificity to particular disorders

**MODEL 1: AUTISM or ASD and schizophrenia as separate disorders**			
MODEL 1a: AUTISM OR ASD ONLY			
	Child	Adult	Comments
CNV is autism risk factor only	Autism or ASD	Autism or ASD; no schizophrenia	ASD traits, but no schizophrenia spectrum traits, in adults
MODEL 1b: SCHIZOPHRENIA ONLY			
	Child	Adult	Comments
CNV is schizophrenia risk factor	Non-clinical or mild premorbidity; no autism or ASD	Schizophrenia; no autism or ASD	Possibe schizophrenia spectrum traits in individuals with CNV but no schizophrenia diagnosis
MODEL 2: ASD and schizophrenia as apparently overlapping disorders			
MODEL 2a: AUTISM OR ASD AND SCHIZOPHRENIA; TRUE OVERLAP			
	Child	Adult	Comments
CNV is risk factor for both autism or ASD and schizophrenia	Autism or ASD	Schizophrenia or autism or ASD	Traits overlapping ASD, autism and schizophrenia, such as sociality and language deficits/abnormalities, are prominent
MODEL 2b: SCHIZOPHRENIA ONLY; AUTISM OR ASD IS FALSE POSITIVE			
	Child	Adult	Comments
CNV is schizophrenia risk factor only; autism or ASD diagnoses are false positives	‘Autism or ASD’ (actually premorbidity to schizophrenia)	Schizophrenia; no autism or ASD	‘ASD’ children have adult relatives with schizophrenia or schizophrenia spectrum; ‘ASD’ is commonly PDD-NOS; child ‘ASD’ phenotypes resemble schizophrenia premorbid phenotypes that are relatively severe

## Methods

CNVs were included for analysis if they met three criteria: (1) previous evidence for statistical association of the CNV with schizophrenia, autism, or both disorders; (2) reports of the CNV in both conditions, from case control, family-based, or case series studies (CNV studies based on ascertainment of a range of childhood conditions, followed by focused analysis on one or more specific CNVs); and (3) sufficient information on phenotypes associated with the CNVs, especially intellectual disability and developmental, speech or language delay, to assess its effects on childhood development, bearing in mind how individuals were ascertained.

Genomic coordinates of the CNVs considered here were defined as in the salient publications (e.g., [21]) and as described below. A pooled analysis was also conducted to test for the focal CNVs as statistically based autism risk factors; this analysis included data from all CNV studies that focused on autism, across multiple loci, using case control or case unaffected sibling designs: Table 1 in Sebat et al. [22], Supplementary Table 2 in Szatmari et al. [23], Table S2 in Christian et al. [24], Table 3 in Marshall et al. [25], Table 1 and pers. comm. in Glessner et al. [26], Supplementary Tables 2, 6, and 8 in Pinto et al. [27], and Table 3 in Sanders et al. [28]. Overlap among studies in autism cases for the AGRE and the AGP data was accounted for in discernment of cases and analysis (i.e., autism and autism spectrum individuals with a given CNV were counted only once). Overall, this analysis included 5,530 cases and 7190 controls. Any overlap between studies in probands who did not harbor CNVs would bias case control analyses against rejection of the null hypothesis (of no association), and thus render the tests performed more conservative. Additional caveats regarding the pooled case control analysis for autism conducted here, and the results from other authors presented in Table 2, include the lack of report of all observed CNVs in some studies [26], possible unobserved overlap among individuals in control groups, and the uses of different technology platforms of varying sensitivities for CNV detection and analysis.

**Table 2:** Data on statistical significance, numbers of cases (in boldface parentheses), and case descriptions, for CNVs that have been reported in both autism and schizophrenia

**CNV**	**Number autism # cases(refs)**^**1**^	**Number controls # cases(refs)**^**1**^	***p*, autism risk, pooled data**^**1**^	***p*, autism risk, prev. studies (# cases) (refs)**	**p, schizo. risk, prev. studies (# cases) (refs)**^**2**^	**Autism, ASD, ID, delays, in case reports, series (refs)**^**3**^	**Model supported**
del 1q21.1	**1**[23]	**0**	0.43	*0.029* (**1**) [4,29]	8.5 × 10^-6^ (**20**)[30]	Yes [29,31]	2b
dup 1q21.1	**7** [23,27,28]	**0**	0.0029	9 × 10^-5^ (**3**)[4,29]	*0.02* (**11**)[30]	Yes [29,31]	2a or 2b
del 3q29	**1**[28]	**0**	0.43		0.0004 (**7**)[30]	Yes [32-34]	2b
del 15q11.2	**2**[27]	**0**	0.19		4.46 × 10^-8^ (**49**)[3]	Yes [35-38]	2b
dup 15q11-q13 (BP2-BP3)	**20** [23,24 26–28]	**0**	<0.0001	4 × 10^-4^, (**6**)[28]; 1 × 10^-5^, (**13**)[26]	0.10 (**2**)[7]	Yes [7,39,40]	1a
del 15q13.3	**3**[27,28]	**0**	0.082		6.9 × 10^-7^ (**21**)[30]	Yes [41-43]	2b
del 16p11.2	**15**[22,25-28]	**4** (26)	0.0015	5 × 10^-29^ (**14**)[28]; 0.044 (**4**)[44]	0.88 (**4**)[30]	Yes [45-48]	1a
dup 16p11.2	**10**[25-28]	**4** (26)	*0.0246*	2 × 10^-5^ (**5**)[28]	2.6 × 10^-8^ (**31**)[30]	Yes [45,47]	2a or 2b
dup 16p13.1	**0**	**0**	ns	*0.023* (**3**)[49]	0.00001 (**12**)[50]	Yes [49,51,52]	2a or 2b
del 17p12	**2**[28]	**0**	0.19	0.58, 0.14 (**5**) [53]^4^	*0.0147* (**4**) [53]	Yes [53-55]	2b
del 22q11.21	**2**[27,28]	**0**	0.19	0.11 (**3**)[28]	7.3 × 10^-13^ (**35**)[30]	Yes [19,56-62]	2b
dup 22q11.21	**9**[24-27]	**0**	0.00055	*0.0218* (**5**)[26]	Not tested in [4] or [30]; too few cases of schizophrenia	Yes [63-70]	1a

See Additional file 1 for details.

^1^ Case control data from Sebat et al. [22], Szatmari et al. [23], Christian et al. [24], Marshall et al. [25], Glessner et al. [26], Pinto et al. [27], and Sanders et al. [28]. Details are provided in Additional file 1.

Fisher's exact test was used.

^2^ Most recent analysis, meta-analysis, or pooled-data analysis.

^3^ Details of these studies are provided in Additional file 1. See also Cooper et al. [71], Kaminsky et al. [21], and Sahoo et al. [72]. "Yes" refers to the presence of autism, ASD, or 'autistic features' among one or more individuals in these reports.

^4^ Tests involve autism cases only, discovery and follow-up samples. See Additional file 1 for details.

## Results

Four of the CNVs, dup 1q21.1, dup 15q11-q13, del 16p11.2, and dup 22q11.21, show clear statistical evidence as autism risk factors, whereas the other eight CNVs considered here, del 1q21.1, del 3q29, del 15q11.2, del 15q13.3, dup 16p11.2, dup 16p13.1, del 17p12, and del 22q11.21, are well supported as risk factors for schizophrenia (Table 2). Three CNVs, dup 1q21.1, dup 16p11.2, and dup16p13.1, exhibit statistical support as risk factors for both autism and schizophrenia, although in each case statistical significance is nominal for one of the two conditions (dup 1q21.1 in schizophrenia, *p* = 0.02; dup 16p11.2 in autism, *p* = 0.025 in the pooled case control analysis; dup 16p13.1 in autism, *p* = 0.023 in the analysis by Table 2 in Hannes et al. [49]). Details regarding all of the relevant studies, for each CNV locus, are presented in Additional file 1.

For all of the CNV risk factors for schizophrenia, case reports and case series demonstrate that multiple children with the CNV have been diagnosed with autism, ASD, autistic features, intellectual disability, developmental, speech, or language delays, and/or multiple congenital anomalies (Table 2 and Additional file 1). Such case studies have generally not, however, evaluated the statistical significance of the CNV as an autism spectrum condition risk factor.

## Discussion

With regard to the predictions of the different models in Table 1, three autism-associated CNVs, dup 15q11-q13, del 16p11.2, and dup 22q11.21, appear to fit with Model 1a, in that very few or no cases of schizophrenia have been reported among individuals with these CNVs and they show a lack of statistical evidence of being schizophrenia risk factors. Evidence regarding the associations of three CNVs, dup 1q21.1, dup 16p11.2, and dup 16p13.1, with autism and schizophrenia risk suggests that they may fit with Model 2a of true overlap, although the nominal nature of one of the statistical associations, for each CNV, indicates that additional data are needed for robust interpretation and exclusion of Model 2b. In addition, for dup 16p13.1, evidence of an association with autism comes from a single analysis that includes only three autism cases with the CNV [49], whereas analysis of the pooled data from seven case control studies provides no evidence of association.

The largest number of CNVs (deletions of 1q21.1, 3q29, 15q11.2, 15q13.3, 17p12, and 22q11.2) appears to fit Model 2b of schizophrenia risk with possible false-positive diagnoses of ASDs in childhood. Such putative false-positive interpretations are based on a combination of four lines of evidence: (1) strong statistical evidence from studies of schizophrenia for the CNV as a risk factor in this disorder; (2) reports of the CNV in some individuals with ASD from large-scale, case control CNV studies of ASD or autism, but with lack of its documentation as a statistically based risk factor for ASD or autism from case control studies; (3) reports of the CNV in ASD or autism cases from case series and case report-based studies that are designed and presented in non-statistical contexts; and (4) the presence, among children with the CNV, of a high frequency of some combination of intellectual disability, developmental delay, or speech or language delay [72], conditions that may represent manifestations of premorbidity to schizophrenia that can contribute to ascertainment and diagnoses of ASDs in children [18,73,74].

Diagnoses of premorbidity to schizophrenia as autism spectrum disorder might be expected in CNV studies for several additional reasons, from previous studies:

1. Schizophrenia involves well-documented premorbidity, in a substantial proportion of cases, which centers on developmental, social, and language deficits [75-78]. In children, premorbidity to schizophrenia most commonly involves ‘negative’ symptoms including deficits in social interaction [79,80], which can overlap with symptoms of autism spectrum disorders [81-85]. Mild to borderline intellectual disability in individuals with schizophrenia is also associated with a higher incidence of negative, compared to positive, symptoms by meta-analysis [86], although this study did not consider effects on premorbid phenotypes. Most of the overlap in symptoms between ASDs and schizophrenia premorbidity appears to involve deficits, delays, or generalized abnormalities, rather than the definitive presence of specific biological or psychological phenotypes. Among all autism spectrum disorders, PDD-NOS is the most commonly diagnosed [87,88]. Such cases usually fail to reach the threshold for autism because of lack of fit to the criteria for restricted interests and repetitive behavior [89], which is also the ASD criterion least likely to be represented in premorbidity to schizophrenia. Premorbidity to schizophrenia is also more severe among males than females [11,80,90], which is concordant with the strong male bias found in autism spectrum disorders [91];

2. Compared to disease-associated SNPs, disease-associated CNVs tend to be rare, more-highly penetrant, syndromic genetic risk factors [8,92,93]. Their deleterious effects on early neurodevelopment might thus be expected to be relatively severe, such that childhood premorbidity, for individuals with schizophrenia-risk CNVs, might be especially prevalent and pronounced compared to individuals without schizophrenia-risk CNVs. A recent study by Sahoo et al. [72] provides evidence consistent with such premorbidity, in that of 38,779 individuals (predominantly children) referred mainly for developmental delay, intellectual disability, autism spectrum disorders, or multiple congenital anomalies, 704 exhibited one of seven CNVs (del 1q21.1, dup 1q21.1, del 15q11.2, del 15q13.3, dup 16p11.2, dup 16p13.11, or del 22q11.2) that has been statistically associated with schizophrenia in studies of adults [30,50].

3. Numerous cases have been reported in the literature of individuals diagnosed with autism spectrum disorders in early to middle childhood, and schizophrenia in late childhood, adolescence, or early adulthood [11,94-100]. Despite these associations, individuals with ASD are apparently not at overall higher risk of later developing schizophrenia [101,102]. Moreover, autism and ASD tend to show familial aggregation of their diagnoses and phenotypes [16,103-105], largely separate from the strong familial aggregation found for schizophrenia [106-108]. A simple hypothesis for explaining these findings would be that sequential diagnoses in the same individuals of ASD (in childhood) and schizophrenia (in adulthood) are sometimes mediated by rare, penetrant schizophrenia risk factors (such as CNVs) that cause relatively severe premorbidity [11]. Direct evidence regarding this hypothesis comes from Addington and Rapoport [109], who showed that individuals with childhood-onset schizophrenia mediated by schizophrenia-associated CNVs were more likely to exhibit premorbidity to schizophrenia in the form of developmental delays (*p* = 0.0001) and may have been more likely to exhibit diagnoses of a PDD (*p* = 0.10) compared to such children lacking these CNVs. Additional evidence comes from studies by Ingason et al. [7] and Chen et al. [110], who each reported sequential diagnoses of autism spectrum disorder in childhood and schizophrenia in adulthood among an individual with such alterations (respectively, a duplication of 15q11-q13 and a balanced translocation disrupting GNB1L, a gene in the 22q11.2 deletion region).

Evaluation of whether ASD diagnoses represent false positives for individuals with schizophrenia-associated CNVs would require longitudinal studies of the individuals involved. Resolution of this question would also benefit from studies of non-clinical individuals bearing risk CNVs, analyses of autism and schizophrenia endophenotypes among individuals with risk CNVs, and studies that focus on the differential diagnosis of autism spectrum disorders in relation to schizophrenia premorbidity in children [11,111-117].

An alternative interpretation to the false-positive hypothesis for diagnoses of ASD among children with schizophrenia-associated CNVs is that such ASD diagnoses are true positives that are compatible with possible later diagnoses of the same individual with schizophrenia [56,99]. Under this interpretation, diagnoses of ASD in children who develop schizophrenia as adolescents or adults would reflect developmental stages, with schizophrenia as the final pathway [118]. By contrast, other ASD cases would fit the classic criteria derived from Kanner [119], Asperger [120], and Rutter [16,17,121], which consider autism as a lifelong condition present from early childhood. Whether or not childhood premorbidity to schizophrenia should be considered an ASD remains an open question, the resolution of which depends in part on the degree to which the causes and phenotypes of ASD in schizophrenia premorbidity overlap with the causes and phenotypes of other cases of ASD.

## Conclusions

One of the primary implications of these results is that hypotheses of autism and schizophrenia sharing substantial genetic etiology with regard to specific CNVs are not strongly supported by the empirical data, and appear to be based in large part on non-statistical evidence from case reports and case series, combining of duplications with deletions for statistical analyses, very small numbers of cases, or pooling of autism or ASD with other diagnoses (such as developmental delay or intellectual disability), which prevents inference concerning which, if any, of the conditions are associated with the focal CNV [1,28,53,122,123]. Additional studies that focus strictly on autism or ASD and schizophrenia for specific CNVs, that take account of childhood premorbidity to schizophrenia as a possible confounding factor, should help to clarify the relationship between these disorders.

## Competing interests

The authors declare that they have no competing interests.

## Authors' Contributions

BC conceived the study and wrote the bulk of the manuscript, and HJC organized and compiled relevant CNV data, and helped in assembling and editing the manuscript. All authors read and approved the final manuscript.

## Supplementary Material

Additional file 1 Association testing of copy number variants in schizophrenia and autism spectrum disorders. Click here for file
