# Supplementary material for: Association testing of copy number variants in schizophrenia and autism spectrum disorders
Source: J Neurodev Disord. 2012 May 30;4(1):15. doi: 10.1186/1866-1955-4-15 (PMC3436704; doi:10.1186/1866-1955-4-15)
Supplement: Additional file 1 — Association testing of copy number variants in schizophrenia and autism spectrum disorders. [file 1866-1955-4-15-S1.doc]

**Association testing of copy number variants in schizophrenia and autism spectrum disorders**

**Bernard Crespi and Helen Crofts**

**Department of Biosciences, Simon Fraser University, Burnaby BC**

**Canada V5A 1S6**

**Supplementary File 1**

Details of data regarding each CNV in Table 2 of main text.

**1q21.1 deletions**

Statistical association between schizophrenia and deletions at 1q21.1 was reported by ISC (2008)(10 deletions in cases, p = 0.046), Stefansson et al. (2008)(Cochran–Mantel–Haenszel test, p = 0.000029), and Kirov et al. (2009) (Fisher’s exact test, p = 2.5 x 10^-8^), who used a pooled sample that included data from ISC, Stefansson et al. (2008) and WTCCC. Levinson et al. (2011) used pooled data from MGS, ISC (2008), and deCODE, showing a strong association of this deletion with schizophrenia (Fisher's exact test, p = 2.2 x 10 ^-8^).

Mefford et al. (2008) reported this deletion in 25 of 5218 patients referred for mental retardation, developmental or speech delays, autism (N = 141), or congenital anomalies; one of these 25 individuals had autism, one had ‘autistic features’, and most of the others (18) showed mental retardation, developmental and/or speech delays. By contrast, the deletion was found in none of 4737 control individuals. The deletion was thus statistically enriched in autism (1 of 141 vs 0 of 4737; Fisher’s exact test, p = 0.0289; analysis in Crespi et al. 2010), though based on a single case. This deletion was not supported as a *de novo* autism risk factor in the analyses of Sanders et al. (2011, Table 4), which used a combined sample from five studies (Sebat et al. 2007; Marshall et al. 2008; Itsara et al. 2010; Pinto et al. 2010; Sanders et al. 2011)(fewer than two deletions reported, across 3816 individuals with autism). This deletion was also unsupported as an autism risk factor in analysis of the pooled data set analyzed here (Table 2), which includes *de novo* and inherited case from seven case-control studies (1 deletion in 5530 cases, none in 7190 controls, Fisher's exact test, p = 0.43). Deletions of 1q21 have been associated with developmental delay, dysmorphic features, and congenital anomalies (Brunetti-Pierri et al. 2008; Mefford et al. 2008).

**1q21.1 duplications**

Levinson et al. (2011) used data from MGS, ISC, and deCODE, to test for an association of this duplication with schizophrenia. In their MGS sample, they reported 7 duplications in 3495 schizophrenia patients compared to none in 3611 controls (Fisher's exact test; p = 0.011); the ISC sample showed 3 duplications in 3391 patients and 1 in 3181 controls (p = 0.27, Fisher's exact test), and the deCODE Iceland sample showed 1 duplication in 648 cases and 12 in 32442 unscreened controls) (p = 0.20, Fisher's exact test); an additional deCODE sample showed no duplications in 579 cases and 1 in 575 controls) (p = 0.50, Fisher's exact test); the pooled sample showed significance at 0.002 by Fisher's exact test and 0.02 by meta-analysis.

Mefford et al. (2008) reported 1q21.1 duplications in three (2.1%) of 141 individuals with autism, and in one (0.02%) of 4737 controls (Fisher’s exact test, p = 0.000092; analysis in Crespi et al. 2010). Duplications of 1q21.1 in autistic individuals have been reported in four other studies (three cases in Szatmari et al. 2007; two cases in Brunetti-Pierri et al. 2008; two cases in Weiss et al. 2008; and one case in Pinto et al. 2010), but in none of these studies were relevant statistical tests performed. This duplication was not supported as a *de novo* autism risk factor in the analyses of Sanders et al. (2011), which used a combined sample from five studies (Sebat et al. 2007; Marshall et al. 2008; Itsara et al. 2010; Pinto et al. 2010; Sanders et al. 2011)(two *de novo* duplications reported among 3816 individuals with autism, p = 0.88). However, analysis of the pooled data set, which includes *de novo* and inherited cases, provides evidence for this duplication as an autism risk factor (seven duplications in 5530 cases, none in 7190 controls, Fisher's exact test, p = 0.0029)(Table 2). As for deletions of 1q21, duplications of this region have been associated with developmental delay, dysmorphic features, and congenital anomalies (Brunetti-Pierri et al. 2008; Mefford et al. 2008).

**3q29 deletions**

Deletions at 3q29 have been associated with schizophrenia by Mulle et al. (2010), using a combined sample of new data and data from four previous studies; overall, six deletions were found among 7545 schizophrenic subjects, and one among 39,748 controls (meta-analysis p = 0.00097; permutation testing p = 0.02; Mulle et al. 2010). Mulle et al. (2010) also reported that one of their deletion patients exhibited ‘mild learning disability’ and ‘impaired social interaction’ as a child, and that an abstract from Quintero-Rivera and Martinez-Agosto (2009, Am. Soc. Human Genet.) described a 10-year old child with the deletion and a history of both autism and schizophrenia. Levinson et al. (2011) used a partially-overlapping sample (with overlap for the ISC data) to also show a significant relationship of 3q29 deletions with schizophrenia (Fisher's exact test, p=0.0004), with five newly-reported deletions in cases. A single deletion in a schizophrenia case individual was also reported by Magri et al. (2010).

Deletions of this region have not been reported in aCGH or SNP-based CNV studies of individuals ascertained for autism or ASD, except for one case in Sanders et al. (2011). This deletion was thus unsupported as an autism risk factor in analysis of the pooled data set, which includes *de novo* and inherited case from seven studies (1 deletion in 5530 cases, none in 7190 controls, Fisher's exact test, p = 0.43)(Table 2). In previous studies, Willat et al. (2005) described six patients with deletions, one of whom was ‘ICD-10 autistic’, and a second who exhibited ‘autistic features’. Ballif et al. (2008) and Clayton-Smith et al. (2010) summarized information on the main clinical features of this deletion, which included, for 19 individuals ascertained for intellectual disability, developmental delay, learning disability, and/or multiple congenital anomalies, mild-moderate learning disability in all 19, speech delay in 11, microcephaly in 11, ‘autistic features’ in five, and depression/psychosis in two, in addition to a facial dysmorphism and various other syndromic phenotypes.

**15q11.2 (BP1-BP2) deletions**

Deletions of the BP1-BP2 region at 15q11.2 were associated with schizophrenia by Stefansson et al. (2008), who reported 26 deletions in 4718 cases, compared to 79 deletions in 41,194 controls (Cochran–Mantel–Haenszel test, p = 0.0006). Kirov et al. (2009) also demonstrated statistically-supported association of this CNV with schizophrenia risk, by pooling data from Stefansson et al. (2008), ISC (2008) and WTCCC (Cardiff) (Fisher's exact test, p = 4.46 x 10 ^-8^)

This CNV has not been statistically associated with autism spectrum disorders, although it has been reported in several studies. Pinto et al. (2010) reported two cases with autism and BP1-BP2 deletions, and Doornbos et al. (2009) reported on nine patients with this CNV (seven of them children). All nine exhibited speech delay, seven showed 'general delay', and four were reported to exhibit ASD (with two of them referred to as 'mild forms'). This deletion was unsupported as an autism risk factor in analysis of the pooled data set, which includes *de novo* and inherited case from seven studies (2 deletions in 5530 cases, none in 7190 controls, FIsher's exact test, p = 0.19)(Table 2).

Burnside et al. (2011) report clinical information on 56 individuals with this deletion, from a large number of children referred for microarray analysis due to 'neurological indications'. Speech delay was reported in 90% (44/49), general developmental delay in 59% (33/56), and 'autism/autistic features/Asperger' in 29% (14/49). von der Lippe et al. (2011) described seven patients with this deletion, from a cohort ascertained for intellectual disability and/or congenital abnormalities. One 24 year-old patient was reported to exhibit psychomotor delay, Asperger syndrome, and paranoid psychosis; a second patient, his brother, was described as similar but without psychosis. None of the other individuals were reported to exhibit psychiatric conditions or phenotypes involving ASD or schizophrenia. Murthy et al. (2007) also described three individuals in one family (a young boy, his half-sister, and their father) with this deletion; the two children exhibited intellectual disability, developmental delay, and speech impairment.

**15q11-q13 duplications**

This region comprises five well-characterized copy-number breakpoints, BP1 to BP5, and BP1-BP2 deletions have been evaluated above (see Sanders et al. 2011, Figure 7). The BP2-BP3 region comprises a suite of imprinted genes, whose deletion results in Prader-Willi syndrome (for paternal deletions) or Angelman syndrome (for maternal deletions). A notable range of duplications and triplications, and other alterations involving the 15q11-q13 region have been reported, some of which have been reported in autistic or ASD individuals (review in Hogart et al. 2010). Of these alterations, the one most-closely considered with regard to autism is maternally-derived interstitial duplications, which involve highly variable phenotypes that most-commonly include intellectual disability, and delays in development, speech and language (Bolton et al. 2001; Hogart et al. 2010). Such duplications, and related abnormalities such as idic(15), have been described as the most common cytogenetic alterations in autism

(e. g, Schroer et al. 1998; Veenstra-Vanderweele et al. 2004), although specification of actual duplication breakpoints is required to define the CNVs involved and statistically evaluate their roles in psychiatric conditions. Paternally-derived duplications of 15q11-q13 are associated with relatively normal, though highly variable, phenotypes compared to the maternal duplications, with several cases of developmental delay, autism or ASD reported (Hogart et al. 2010).

Duplications of 15q11-q13 have been reported in several CNV studies of autism, including Sebat et al. (2007; 1 in 195 autism cases), Christian et al. (2008, 3 in 397 cases), Marshall et al. (2008, 2 in 427 families), Bucan et al. (2009, 6 in 912 families) and Glessner et al. (2009, 8 in 859 cases, ACC data; AGRE data shows overlap with other studies), but these duplications vary in size from about 5 to12Mb and only Christian et al. (2008) reported the parental origin (maternal) of the duplications.

Two studies have provided statistical analysis of 15q11-q13 duplications of BP2-BP3 in autism: (1) Glessner et al. (2009), who showed that a CNV spanning the BP2-BP3 region was statistically enriched in a combined AGRE and ACC sample (13 unrelated cases overall; p = 1.0 x 10 ^-5^), and (2) Sanders et al. (2011), who showed that *de novo* BP2-BP3 duplications were enriched in autism for a combined sample from five studies (Sebat et al. 2007; Marshall et al. 2008; Itsara et al. 2010; Pinto et al. 2010; Sanders et al. 2011)(six duplications reported among 3816 individuals with autism, p = 4 X 10 ^-4^, maternal vs. paternal status not reported). Analysis of the pooled data set, which includes *de novo* and inherited cases from seven studies, provides strong evidence for this duplication as an autism risk factor (20 deletions in 5530 cases, none in 7190 controls, Fisher's exact test, p < 0.0001)(Table 2). Among these 20 deletions, parental origin (maternal) was verified for only three.

Ingason et al. (2011a) recently described evidence regarding the incidence of maternally-derived duplications at 15q11-q13 in psychotic illness, showing such duplications in 4 of 7582 patients with schizophrenia or schizoaffective disorder, compared to 3 in 41,370 controls (Fisher's exact test, p = 0.012 in their analysis). However, of the four patients in this test with maternal duplications, one had a duplication that spanned BP1-BP3, two showed duplications of BP2-BP3, and one had a duplication from BP1 to a region between BP4 and BP5; by contrast, of the comparison subjects with maternal duplications, two had BP1-BP3 duplications and one had a duplication of BP2-BP3. Restricting the analysis to the BP2-BP3 region analyzed by Glessner et al. (2010) and Sanders et al. (2011) for autism (and noting that BP1-BP2 region deletions have been associated statistically with schizophrenia), yields two patients and one control with the maternal duplication, which represents a non-significant difference in frequency (Fisher’s Exact test, p = 0.061). If the BP2-BP3 duplication is considered regardless of parental origin (as in Sanders et al., 2011, and in the pooled analysis for autism described above and Table 2), then two patients and two controls show the duplications (Fisher's exact test, P = 0.10).

Ingason et al. (2011a) also noted that intellectual disability and developmental delays were frequent in the individuals harboring these CNVs, that all of the case patients had family histories of psychotic-affective illnesses, and that one of the case patients was diagnosed with early childhood autism prior to a diagnosis of schizophrenia.

**15q13.3 deletions**

This deletion involves BP4-BP5 in the 15q11-q13 region. A statistical association between 15q13.3 deletions and schizophrenia was reported by Stefansson et al. (2008*)*(Cochran–Mantel–Haenszel test, p = 0.00053), by ISC (2008) (nine deletions in cases, p = 0.046) and by Kirov et al. (2009) in their pooled sample (Fisher's exact test, p = 2.8 x 10 ^-8^). Levinson et al. (2011) used data from MGS, ISC (2008), and deCODE, to test for an association of this deletion with schizophrenia. In their MGS sample, they reported 7 deletions in 3495 schizophrenia patients compared to 1 in 3611 controls (Fisher's exact test, p = 0.04); the ISC sample showed 8 deletions in 3391 patients and none in 3181 controls (Fisher's exact test, p = 0.005), and the pooled deCODE sample showed 5 deletions in 3550 cases and 8 in 43130 controls) (Fisher's exact test, p = 0.0017); pooled analysis showed p = 6.9 x 10 ^-7^ by meta-analysis and p=2.0 x 10 ^-9^ by Fisher's exact test.

Deletions at 15q13.3 have not been statistically associated with autism in CNV or other studies. Although children with autism spectrum disorders and the deletion have been reported in several studies, including Pinto et al. (2010)(1 case) and Sanders et al. (2011)(2 cases), this deletion was unsupported as an autism risk factor in analysis of the pooled data set, which includes *de novo* and inherited case from seven studies

(3 deletions in 5530 cases, none in 7190 controls, Fisher's exact test, p = 0.082)(Table 2).

Miller et al. (2008) reported five cases of this deletion among 1445 children or adolescents referred for autism spectrum disorders, mental retardation, developmental delay, learning disability, multiple congenital anomalies, dysmorphic features, seizures, or other phenotypes; two of them were given diagnoses of PDD-NOS, one showed ‘autistic features’, and one had diagnoses of bipolar disorder and anxiety disorder (at age 10). Ben-Shachar et al. (2009) identified 20 individuals (including 14 children and 6 parents, among 12 families) with this deletion, from about 8200 individuals referred for testing by similar criteria used in Miller et al. (2008). Children in four of the 12 families showed autism spectrum disorders, including 1 case of Asperger’s, 2 MZ twins with autism, and 2 cases with ‘autism disorder’. Among parents in the 12 families, two fathers with the deletion had bipolar disorder, one father had schizophrenia (with deletion studies unknown), and one mother had ‘mental illness’. Van Bon et al. (2009) described 17 individuals with deletions of BP3-BP5 or BP4-BP5, noting that all but one of them showed some degree of cognitive impairment, from mild learning problems to severe mental retardation; one patient with a BP4-BP5 deletion also exhibited 'autistic features'. The only additional cases of children with autism and this deletion are a ‘control’ individual described in Stefansson et al. (2008), and a case in Pinto et al. (2010).

**16p11.2 deletions**

Deletions of 16p11.2 have not been associated with schizophrenia. McCarthy et al. (2009) reported the same frequency of 16p11.2 deletions (0.3%) in schizophrenia patients as in controls. Similarly, Levinson et al. (2011) used pooled data from the MGS, ISC, deCODE, Weiss et al. (2008), and McCarthy et al. (2009) studies, and found a lack of association of 16p11.2 deletions with schizophrenia (p > 0.50).

Kumar et al. (2008) reported a statistical association of autism with deletions at 16p11.2, with deletions found in 4 of 712 autism cases from AGRE and NIMH and no deletions in 837 controls (Fisher’s exact test, p = 0.044). This finding was also supported by combining Kumar’s data with data from the (non-overlapping) ACC population of Glessner et al. (2009) (Fisher’s exact test, p = 0.0295, analysis in Crespi et al. 2010). Deletions plus duplications were significantly associated with autism spectrum disorder in data from Weiss et al. (2008) (3 of 751 families in an AGRE sample and 3 individuals in a deCODE sample); a Children’s Hospital Boston sample in Weiss et al. (2008) was ascertained for 'developmental delay, mental retardation, or autism spectrum disorders', and thus cannot be used for evaluating associations with ASD in particular. Deletions were also found by McCarthy et al. (2010) at a significantly higher frequency in 'autism or global developmental delay' than in controls, using pooled data from their study, Weiss et al. (2008), Marshall et al. (2008), and ISC (2008). *De novo* deletions were also found to be enriched in autism for a combined sample from five studies (Sebat et al. 2007; Marshall et al. 2008; Itsara et al. 2010; Pinto et al. 2010; analysis in Sanders et al. 2011)(14 deletions reported among 3816 individuals with autism, p = 5 X 10^-29^). Analysis of the pooled data set, which includes *de novo* and inherited cases from seven studies, also provides strong evidence for this deletion as an autism risk factor (15 deletions in 5530 cases, 4 deletions in 7190 controls, Fisher's exact test, p = 0.00147)(Table 2).

Shinawi et al. (2010) describe evidence for high rates of speech/language delay and cognitive impairment (14 of 14 patients) in children with 16p11.2 deletions, and 3 of 11 patients with the deletion were diagnosed with autism spectrum disorder. Similarly, Bijlsma et al. (2009) found that 14 (0.33%) of 4284 individuals referred for mental retardation and/or multiple congenital anomalies carried deletions of 16p11.2. Fernandez et al. (2009) reported detailed phenotypic information on three families with the CNV; all three probands exhibited ASD or autism. McCarthy et al. (2009) presents evidence of significantly larger head circumference in patients with the deletion than with the duplication, Shinawai et al. (2010) describe statistically significant macrocephaly among their patients with the deletion, and Jacquemont et al. (2011) found significantly higher head circumference in individuals with deletions than in controls. Hanson et al. (2010) report varying levels of intellectual disability, a high incidence of language delay, and high rates of autism (9 of 11 cases, by ADOS, ADI, or both) among individuals with this deletion. Similarly, Rosenfeld et al. (2010) reported, among 18 individuals with the microdeletion, developmental delay in all, speech or language problems in most, and three with ASD (PDD-NOS) among the 16 old enough to be evaluated.

**16p11.2 duplications**

McCarthy et al. (2009) provided evidence, from a discovery data set (Cochran-Mantel-Haenszel test, p = 0.000014), a replication data set (Cochran-Mantel-Haenszel test, p = 0.022), and a meta-analysis (p = 4.8 x 10 -7, p = 0.0000005) for association of duplications at 16p11.2 with schizophrenia, and Glessner et al. (2010) also showed enrichment of this CNV in schizophrenia (duplications in 13 cases and 1 control, Fisher's exact test, p = 5.7 x 10 ^-6^). This result was also supported by analyses in Levinson et al. (2011), who used pooled data from the MGS, ISC, deCODE, Weiss et al. (2008), and McCarthy et al. (2009) studies (Fisher's exact test p = 1.5 x 10 ^-12^).

Weiss et al. (2008) reported 16p11.2 duplications in three of 751 AGRE families, and in four additional individuals from among 512 children ascertained for developmental delay, mental retardation, or autism spectrum disorder. They reported a significant association of deletions or duplications (pooled) with ASD in the AGRE sample (p = 1.1 x 10 ^-4^) but did not analyze duplications in particular. Their deCODE sample includes no duplications in 299 autism spectrum cases, compared to 5 duplications in 18834 unscreened controls (n. s.). Data from Glessner et al*.* 2009) included four duplications in 2195 cases (three of which are the same as those in Weiss’s AGRE sample), and three duplications in 2519 controls (p = 0.425, Fisher's exact test). McCarthy et al. (2009) provided evidence for significantly higher rates of 16p11.2 duplications in 'autism or global developmental delay' using pooled data from their study, Weiss et al. (2008), Marshall et al. (2008), and ISC (2008), but did not report results for autism or ASD in particular. *De novo* duplications were, however, found to be enriched in autism for a combined sample from five studies (Sebat et al. 2007; Marshall et al. 2008; Itsara et al. 2010; Pinto et al. 2010; analysis in Sanders et al. 2011)(5 duplications reported among 3816 individuals with autism, p = 2 X 10 ^-5^). Analysis of the pooled data set, which includes *de novo* and inherited cases from seven studies, provides nominal evidence for this duplication as an autism risk factor (10 duplications in 5530 cases, 4 in 7190 controls, Fisher's exact test, p = 0.0246)(Table 2).

Shinawi et al. (2010) describe evidence for high rates of speech/language delay and cognitive impairment (10 of 10 patients) in children with 16p11.2 duplications; none of 10 patients were diagnosed with autism, but 2 had 'autistic features'. Fernandez et al. (2009) reported detailed phenotypic information on three families with this duplication; two probands exhibited ASD or autism, and one had developmental delay; the duplication-posiitve father of the latter proband was diagnosed with bipolar disorder. As noted above, McCarthy et al. (2009) presents evidence of significantly larger head circumference in patients with the deletion than with the duplication; Shinawai et al. (2010) report that six of 10 duplication patients exhibited microcephaly, and Jacquemont et al. (2011) found significantly lower head circumference in individuals with duplications than in controls Rosenfeld et al. (2010) reported, among 19 individuals with the microduplication, developmental, speech, or language delays in the majority.

**16p13.1 duplications**

Ingason et al. (2011b) reported an association of 16p13.1 duplications with schizophrenia, for a region spanning two duplication intervals (I and II, p = 0.00001), and for duplications across the three intervals considered overall (p = 0.007). These relationships held for males and for both sexes pooled, but not among females, and four of the schizophrenia cases with duplications showed relatively early ages of onset (12,17,19 and 19 years). Duplications of interval II were not supported as schizophrenia risk factors, with no cases reported in schizophrenic individuals, but six duplications among 34,421 controls (Ingason et al. 2011b, Table 1).

Ullmann et al. (2007) reported duplications of interval I + II in three (1.65%) of 182 unrelated individuals with autism (all male), and noted a lack of this duplication in ‘over 600 normal individuals and patients from other cohorts’. They also noted that some family members of these three cases were only mildly affected, or apparently unaffected, by the presence of the duplication.

Duplications of 16p13.1 have been reported in two other studies of CNVs in autism. Sanders et al. (2011) found two duplications (of interval II) among 1124 individuals with autism (as well as four duplications in siblings), and Pinto et al. (2010) reported two duplications (also of interval II) among 875 individuals with autism. The lack of any reports of 16p13.1 duplications of interval I + II in any other studies that focussed on autism suggests that data in the Ullman et al. (2007) may not be representative of other sets of individuals with autism.

Hannes et al. (2008) found five duplications of intervals I + II among 1027 patients with mental retardation and/or multiple congenital anomalies; they also reported the duplication in 5 of 2014 controls, such that the duplication was not statistically enriched in patients; three of the five duplication patients exhibited mental retardation, one showed developmental delay, and one had speech delay, but none was reported with an ASD. However, Hannes et al. (2008) also noted that if the Ullman et al. (2007) patients data (3/182) is combined with their data on controls (5/2104), then the duplication appears to be enriched in autism (Fisher’s exact test, p = 0.023).

Ramalingam et al. (2011) screened 1645 patients referred for developmental delay, ASD, seizures, dysmorphic features, or multiple congenital anomalies for CNVs, finding five individuals with duplications of the I+II interval. Three of these five individuals were reported to exhibit ASD, one showed developmental delay, and one had generalized epilepsy. The absence of data on what proportion of the patients population exhibited ASD, and the lack of a designated control population for this study, precludes statistical testing of this association.

**17q12 deletions**

Moreno-De-Luca et al. (2010) describe evidence relating deletions at 17q12 to schizophrenia and autism. Schizophrenia data from the SGENE and GAIN studies were analyzed, with pooled samples showing 4 of 6340 cases with the deletion, compared to none of 43,076 controls (Cochran-Mantel-Haenszel test, p = 0.0147).

For their ‘autism’ or 'ASD’ cases, Moreno-DeLuca et al. (2010) used four samples of patients with different sets of diagnoses: (1) ‘ASD, developmental delay, and/or intellectual disability’ (ISCA Consortium); (2) ‘developmental delay (autistic spectrum disorders, intellectual disability, or slow expressive language development)’ (Icelandic sample); (3) ‘ASD’ (Simons Simplex Collection); and (4) nine index patients available for detailed clinical assessment, six of whom exhibited ‘autism or autistic features’, and four of whom were formally diagnosed with autism. Their combined discovery patient sample of individuals with autism, developmental delay, and/or intellectual disability showed 18 of 15749 cases with the deletion, compared to no deletions in 4519 controls (Cochran-Mantel-Haenszel test, p = 0.0196); a follow-up cohort from SSC and Iceland showed 2 deletions in 1182 cases, compared to none in 38,498 controls (Cochran-Mantel-Haenszel test, p = 0.00192).

Across all patients with the 17q12 deletion, five (1 from SSC, 4 from ISCA) were known to be diagnosed with autism (by DSM-IV and/or ADI-ADOS), in comparison to the other conditions. In case-control comparisons, analysis of these data indicates no evidence for association of 17q12 deletion with autism *per se* in the Discovery sample (4/15,763 vs 0/4519; Fisher’s exact test, p = 0.58), or in the follow-up SSC sample (1/806 vs 0/4853, Fisher's exact test, p = 0.14); no cases of autism in 17q12 deletion were verified in the Icelandic sample. Moreno-DeLuca et al. thus demonstrate that 17q12 deletion is statistically associated with a combined set of three categories (autism, developmental delay, and intellectual disability) but analysis of their data provides no evidence for association with autism or ASD. As regards association with autism, analysis of the pooled data set, which includes *de novo* and inherited cases from seven studies, provides no evidence for this deletion as an autism risk factor (2 deletions in 5530 cases, none in 7190 controls, Fisher's exact test, p = 0.189)(Table 2).

Five of the nine index patients described in Moreno-DeLuca et al. (2010) exhibited phenotypes that have been associated with schizophrenia or affective conditions, which included bipolar disorder, depression, ‘mood changes’, unusual affect, flat affect, and irritability (their Table 1 and Supplementary information); only three of these individuals (exhibiting the former four phenotypes) were over 18 years of age. Seven of these nine individuals showed speech and/or developmental delays.

Deletions of this region of 17q12 are notably syndromic and involve abnormalities of the kidney, pancreas, and liver (Nagamani et al. 2010). Nagamani et al. (2010) reported that 3 of 5 of their patients with 17q12 deletions also exhibited ‘central nervous system involvement’, including (1) receptive and expressive speech problems, (2) speech delay, and (3) moderate-to-severe mental retardation. Loirat et al. (2010) described 3 children with 17q12 deletions, severe developmental delays, and autism diagnoses by ADI-R, of 53 children ascertained for kidney dysfunction. However, deletions or duplications of this region of 17q12 have not been reported in studies of individuals ascertained for autism or ASD, except for two cases reported in Sanders et al. (2011), which did not indicate statistically increased frequency in autism.

**22q11.2 deletions**

The 1.5 Mb and 3 Mb deletions at 22q11.2 have been determined cytogenetically since the late 1990s and they represent well-documented, highly-penetrant risk factors for schizophrenia, with 25-30% of subjects exhibiting the disorder in adulthood (Bassett et al. 2008; Prasad et al. 2008). Statistical associations between 22q11.2 deletions and schizophrenia have been reported by ISC (2008) (11 deletions in cases, empirical p = 0.0046), and Glessner et al. (2010)(10 deletions in cases, Fisher’s exact test, p = 1.6 x 10 ^-5^). Levinson et al. (2011) analyzed pooled data from MGS (their study), ISC, and deCODE, and found a strong association (35 total cases, Fisher's exact test, p < 1.0 x 10 ^-16)^ of the deletion with schizophrenia.

CNV studies of autism spectrum disorders provide no support for association of 22q11.2 deletions with ASD. Sanders et al. (2011, Table 4) reported three *de novo* cases in a combined sample from five studies (Sebat et al. 2007; Marshall et al. 2008; Itsara et al. 2010; Pinto et al. 2010; Sanders et al. 2011)(3816 individuals with autism,

p = 0.11). Analysis of the pooled data set, which includes *de novo* and inherited cases from seven case-control studies of autism, provides also provides no evidence for this deletion as an autism risk factor (2 deletions in 5530 cases, none in 7190 controls, Fisher's exact test, p = 0.189)(Table 2). Ogilvie et al. (2000) reported no cases of 22q11.21 deletion in a sample of 103 subjects diagnosed with autism by strict criteria.

Autistic spectrum disorders have been diagnosed at high rates (up to 20% for autistic disorder, and 40-50% for PDD or a broad autism phenotype) in children with 22q11.2 deletions in several studies (Fine et al. 2005; Vorstman et al. 2006; Antshel et al. 2007; Kates et al. 2007; Niklasson et al. 2009). Eliez (2007) and Feinstein and Singh (2007) argued that diagnoses of autism in children and adolescents with this deletion appear to represent false positives, based on the differing cognitive-behavioral profiles of individuals with 22q11.2 deletion compared to idiopathic autism, and the overlap of ‘autistic’ traits, even as defined by metrics such as ADI-R, with traits characteristic of premorbidity to schizophrenia. In response to Eliez (2007), Vorstman et al. (2007) agreed that autism and autistic symptoms in 22q11.21 deletion may indeed represent prepsychotic psychological difficulties, but that diagnoses of autism spectrum disorders remained valid in such cases. Gothelf et al. (2008) suggested that high rates of autistic disorder in neurogenetic syndromes such as 22q11.2 deletion may represent a ‘pitfall’ of DSM criteria that were not designed for assessing deficits in social phenotypes in the context of reduced overall levels of cognitive function.

Development in individuals with 22q11.2 deletions is characterized by motor, language and speech delays in almost all individuals, mild to moderate intellectual diability in about half of children, and a wide range of congenital malformations (Murphy and Scambler 2005; Gothelf 2007)

**22q11.2 duplications**

Reciprocal duplications of the 22q11.2 intervals show no evidence of association with schizophrenia in CNV studies (with one duplication reported among the eight schizophrenia studies analyzed in Crespi et al. 2010). Brunet et al. (2008) tested 190 patients with schizophrenia for 22q11.2 copy number alterations, reporting two cases of deletions, but no duplications, and Levinson et al. (2011) did not include this CNV in their analyses, due to lack of evidence.

A hypothesis of statistical association of 22q11.2 duplications with autism was tested by Glessner et al. (2009), who reported a total of five duplications (meeting the 1.5Mb criterion; Glessner, pers. comm.) in 2195 cases, compared to none in 2519 controls (Fisher’s exact test, p = 0.0218). Data summarized by Crespi et al. (2010) show 22q11.2 duplications among eight individuals with autism, across four CNV studies; one duplication was also reported in Bucan et al. (2009) and two duplications were found in Pinto et al. (2010). Case reports and case series of autism in 22q11.2 duplication are described in Lo-Castro et al. (2009), Hassad et al. (2004), Mukades and Herguner (2007) and Ramelli et al. (2008), and Chen et al. (2011) reported duplications in two families with autism. This duplication was not supported as a *de novo* autism risk factor in the analyses of Sanders et al. (2011), which used a combined sample from five studies (Sebat et al. 2007; Marshall et al. 2008; Itsara et al. 2010; Pinto et al. 2010; Sanders et al. 2011), finding two duplications reported among 3816 individuals with autism. However analysis of the pooled data set, which includes *de novo* and inherited cases from seven studies, provides strong evidence for this duplication as an autism risk factor (9 duplications in 5530 cases, none in 7190 controls, Fisher's exact test, p = 0.00055)(Table 2).

Phenotypes associated with 22q11.2 duplication are milder and more variable than those reported in the deletion, although developmental delay, speech delay, and cognitive deficits have each been reported in 50-80% of patients (Ensenauer et al. 2003; Courtens et al. 2008; Ou et al. 2008; Portnoi 2009). This CNV appears to be inherited more frequently than the reciprocal deletion (Ou et al. 2008), and its incidence may be higher than case reports indicate, due to the presence of individuals with the duplication and normal and near-normal phenotypes (Courtens et al. 2008).

**References**

Antshel KM, Aneja A, Strunge L, Peebles J, Fremont WP, Stallone K, et al. Autistic spectrum disorders in velo-cardio facial syndrome (22q11.2 deletion). J Autism Dev Disord. 2007;37(9):1776-86.

Ballif BC, Theisen A, Coppinger J, Gowans GC, Hersh JH, Madan-Khetarpal S, et al. Expanding the clinical phenotype of the 3q29 microdeletion syndrome and characterization of the reciprocal microduplication. Mol Cytogenet. 2008;1:8.

Bassett AS, Scherer SW, Brzustowicz LM. Copy number variations in schizophrenia: critical review and new perspectives on concepts of genetics and disease. Am J Psychiatry. 2010;167(8):899-914.

Ben-Shachar S, Lanpher B, German JR, Qasaymeh M, Potocki L, Nagamani SC, et al. Microdeletion 15q13.3: a locus with incomplete penetrance for autism, mental retardation, and psychiatric disorders. J Med Genet. 2009;46(6):382-8.

Bijlsma EK, Gijsbers AC, Schuurs-Hoeijmakers JH, van Haeringen A, Fransen van de Putte DE, Anderlid BM, et al. Extending the phenotype of recurrent rearrangements of 16p11.2: deletions in mentally retarded patients without autism and in normal individuals. Eur J Med Genet. 2009;52(2-3):77-87.

Bolton PF, Dennis NR, Browne CE, Thomas NS, Veltman MW, Thompson RJ, et al.The phenotypic manifestations of interstitial duplications of proximal 15q with special reference to the autistic spectrum disorders. Am J Med Genet. 2001;105(8):675-85.

Brunet A, Armengol L, Pelaez T, Guillamat R, Vallès V, Gabau E, et al. Failure to detect the 22q11.2 duplication syndrome rearrangement among patients with schizophrenia. Behav Brain Funct. 2008;;4:10.

Brunetti-Pierri N, Berg JS, Scaglia F, Belmont J, Bacino CA, Sahoo T, et al. Recurrent reciprocal 1q21.1 deletions and duplications associated with microcephaly or macrocephaly and developmental and behavioral abnormalities. Nat Genet. 2008;40(12):1466-71.

Bucan M, Abrahams BS, Wang K, Glessner JT, Herman EI, Sonnenblick LI, et al. Genome-wide analyses of exonic copy number variants in a family-based study point to novel autism susceptibility genes. PLoS Genet. 2009;5(6):e1000536.

Burnside RD, Pasion R, Mikhail FM, Carroll AJ, Robin NH, Youngs EL, et al. Microdeletion/microduplication of proximal 15q11.2 between BP1 and BP2: a susceptibility region for neurological dysfunction including developmental and language delay. Hum Genet. 2011 (in press).

Christian SL, Brune CW, Sudi J, Kumar RA, Liu S, Karamohamed S, et al. Novel submicroscopic chromosomal abnormalities detected in autism spectrum disorder. Biol Psychiatry. 2008;63(12):1111-7.

# [Chen YZ](http://www.ncbi.nlm.nih.gov/pubmed?term="Chen YZ"%5BAuthor%5D), [Matsushita M](http://www.ncbi.nlm.nih.gov/pubmed?term="Matsushita M"%5BAuthor%5D), [Girirajan S](http://www.ncbi.nlm.nih.gov/pubmed?term="Girirajan S"%5BAuthor%5D), [Lisowski M](http://www.ncbi.nlm.nih.gov/pubmed?term="Lisowski M"%5BAuthor%5D), [Sun E](http://www.ncbi.nlm.nih.gov/pubmed?term="Sun E"%5BAuthor%5D), [Sul Y](http://www.ncbi.nlm.nih.gov/pubmed?term="Sul Y"%5BAuthor%5D), [Bernier R](http://www.ncbi.nlm.nih.gov/pubmed?term="Bernier R"%5BAuthor%5D), [Estes A](http://www.ncbi.nlm.nih.gov/pubmed?term="Estes A"%5BAuthor%5D), [Dawson G](http://www.ncbi.nlm.nih.gov/pubmed?term="Dawson G"%5BAuthor%5D), [Minshew N](http://www.ncbi.nlm.nih.gov/pubmed?term="Minshew N"%5BAuthor%5D), [Shellenberg GD](http://www.ncbi.nlm.nih.gov/pubmed?term="Shellenberg GD"%5BAuthor%5D), [Eichler EE](http://www.ncbi.nlm.nih.gov/pubmed?term="Eichler EE"%5BAuthor%5D), [Rieder MJ](http://www.ncbi.nlm.nih.gov/pubmed?term="Rieder MJ"%5BAuthor%5D), [Nickerson DA](http://www.ncbi.nlm.nih.gov/pubmed?term="Nickerson DA"%5BAuthor%5D), [Tsuang DW](http://www.ncbi.nlm.nih.gov/pubmed?term="Tsuang DW"%5BAuthor%5D), [Tsuang MT](http://www.ncbi.nlm.nih.gov/pubmed?term="Tsuang MT"%5BAuthor%5D), [Wijsman EM](http://www.ncbi.nlm.nih.gov/pubmed?term="Wijsman EM"%5BAuthor%5D), [Raskind WH](http://www.ncbi.nlm.nih.gov/pubmed?term="Raskind WH"%5BAuthor%5D), [Brkanac Z](http://www.ncbi.nlm.nih.gov/pubmed?term="Brkanac Z"%5BAuthor%5D). Evidence for involvement of GNB1L in autism. Am J Med Genet B Neuropsychiatr Genet. 2011 (in press).

Clayton-Smith J, Giblin C, Smith RA, Dunn C, Willatt L. Familial 3q29 microdeletion syndrome providing further evidence of involvement of the 3q29 region in bipolar disorder. Clin Dysmorphol. 2010;19(3):128-32.

Courtens W, Schramme I, Laridon A. Microduplication 22q11.2: a benign polymorphism or a syndrome with a very large clinical variability and reduced penetrance?--Report of two families. Am J Med Genet A. 2008;146A(6):758-63.

Crespi B, Stead P, Elliot M. Comparative genomics of autism and schizophrenia. Proc Natl Acad Sci U S A. 2010;107 Suppl 1:1736-41.

Doornbos M, Sikkema-Raddatz B, Ruijvenkamp CA, Dijkhuizen T, Bijlsma EK, Gijsbers AC, et al. Nine patients with a microdeletion 15q11.2 between breakpoints 1 and 2 of the Prader-Willi critical region, possibly associated with behavioural disturbances.. Eur J Med Genet. 2009 Mar-Jun;52(2-3):108-15.

Eliez S. Autism in children with 22q11.2 deletion syndrome. J Am Acad Child Adolesc Psychiatry. 2007;46(4):433-4.

Ensenauer RE, Adeyinka A, Flynn HC, Michels VV, Lindor NM, Dawson DB, et al. Microduplication 22q11.2, an emerging syndrome: clinical, cytogenetic, and molecular analysis of thirteen patients. Am J Hum Genet. 2003;73(5):1027-40.

Feinstein C, Singh S. Social phenotypes in neurogenetic syndromes. Child Adolesc Psychiatr Clin N Am. 2007;16(3):631-47.

Fernandez BA, Roberts W, Chung B, Weksberg R, Meyn S, Szatmari P, et al. Phenotypic spectrum associated with de novo and inherited deletions and duplications at 16p11.2 in individuals ascertained for diagnosis of autism spectrum disorder. J Med Genet. 2010;47(3):195-203.

Fine SE, Weissman A, Gerdes M, Pinto-Martin J, Zackai EH, McDonald-McGinn DM, et al. Autism spectrum disorders and symptoms in children with molecularly confirmed 22q11.2 deletion syndrome. J Autism Dev Disord. 2005;35(4):461-70.

Glessner JT, Wang K, Cai G, Korvatska O, Kim CE, Wood S, et al. Autism genome-wide copy number variation reveals ubiquitin and neuronal genes. Nature. 2009;459(7246):569-73.

Glessner JT, Reilly MP, Kim CE, Takahashi N, Albano A, Hou C, et al. Strong synaptic transmission impact by copy number variations in schizophrenia. Proc Natl Acad Sci U S A. 2010;107(23):10584-9.

Gothelf D. Velocardiofacial syndrome. Child Adolesc Psychiatr Clin N Am. 2007;16(3):677-93.

Gothelf D, Schaer M, Eliez S. Genes, brain development and psychiatric phenotypes in velo-cardio-facial syndrome. Dev Disabil Res Rev. 2008;14(1):59-68.

Hannes FD, Sharp AJ, Mefford HC, de Ravel T, Ruivenkamp CA, Breuning MH, et al. Recurrent reciprocal deletions and duplications of 16p13.11: the deletion is a risk factor for MR/MCA while the duplication may be a rare benign variant. J Med Genet. 2009;46(4):223-32.

Hanson E, Nasir RH, Fong A, Lian A, Hundley R, Shen Y, et al. Cognitive and behavioral characterization of 16p11.2 deletion syndrome. J Dev Behav Pediatr. 2010;31(8):649-57.

Hassed, S., S. A. Vaz, J. Lee, J. J. Mulvihill, and S. Li. 2004. Expanded phenotype of the 22q duplication syndrome. American Journal of Human Genetics 75(Suppl.):151.

Hogart A, Wu D, LaSalle JM, Schanen NC. The comorbidity of autism with the genomic disorders of chromosome 15q11.2-q13. Neurobiol Dis. 2010;38(2):181-91.

International Schizophrenia Consortium. Rare chromosomal deletions and duplications increase risk of schizophrenia. Nature. 2008;455(7210):237-41.

Ingason A, Kirov G, Giegling I, Hansen T, Isles AR, Jakobsen KD, et al. Maternally derived microduplications at 15q11-q13: implication of imprinted genes in psychotic illness. Am J Psychiatry. 2011a;168(4):408-17.

Ingason A, Rujescu D, Cichon S, Sigurdsson E, Sigmundsson T, Pietiläinen OP, et al. Copy number variations of chromosome 16p13.1 region associated with schizophrenia. Mol Psychiatry. 2011b;16(1):17-25.

Itsara A, Cooper GM, Baker C, Girirajan S, Li J, Absher D, et al. Population analysis of large copy number variants and hotspots of human genetic disease. Am J Hum Genet. 2009;84(2):148-61.

Jacquemont S, Reymond A, Zufferey F, Harewood L, Walters RG, Kutalik Z, et al. Mirror extreme BMI phenotypes associated with gene dosage at the chromosome 16p11.2 locus. Nature. 2011 Aug 31.

Kates WR, Antshel KM, Fremont WP, Shprintzen RJ, Strunge LA, Burnette CP, et al. Comparing phenotypes in patients with idiopathic autism to patients with velocardiofacial syndrome (22q11 DS) with and without autism. Am J Med Genet A. 2007;143A(22):2642-50.

Kirov G, Grozeva D, Norton N, Ivanov D, Mantripragada KK, Holmans P et al. Support for the involvement of large copy number variants in the pathogenesis of schizophrenia. Hum Mol Genet. 2009;18(8):1497-503.

Kumar RA, KaraMohamed S, Sudi J, Conrad DF, Brune C, Badner JA, et al. Recurrent 16p11.2 microdeletions in autism. Hum Mol Genet. 2008;17(4):628-38.

Levinson DF, Duan J, Oh S, Wang K, Sanders AR, Shi J, et al. Copy number variants in schizophrenia: confirmation of five previous findings and new evidence for 3q29 microdeletions and VIPR2 duplications. Am J Psychiatry. 2011;168(3):302-16.

Lo-Castro A, Galasso C, Cerminara C, El-Malhany N, Benedetti S, Nardone AM, et al. Association of syndromic mental retardation and autism with 22q11.2 duplication. Neuropediatrics. 2009;40(3):137-40.

Loirat C, Bellanné-Chantelot C, Husson I, Deschênes G, Guigonis V, Chabane N. Autism in three patients with cystic or hyperechogenic kidneys and chromosome 17q12 deletion. Nephrol Dial Transplant. 2010;25(10):3430-3.

Magri C, Sacchetti E, Traversa M, Valsecchi P, Gardella R, Bonvicini C, et al. New copy number variations in schizophrenia. PLoS One. 2010;5(10):e13422.

Marshall CR, Noor A, Vincent JB, Lionel AC, Feuk L, Skaug J, et al. Structural variation of chromosomes in autism spectrum disorder. Am J Hum Genet. 2008;82(2):477-88.

McCarthy SE, Makarov V, Kirov G, Addington AM, McClellan J, Yoon S, et al. Microduplications of 16p11.2 are associated with schizophrenia. Nat Genet. 2009;41(11):1223-7.

Mefford HC, Sharp AJ, Baker C, Itsara A, Jiang Z, Buysse K, et al. Recurrent rearrangements of chromosome 1q21.1 and variable pediatric phenotypes. N Engl J Med. 2008;359(16):1685-99.

Miller DT, Shen Y, Weiss LA, Korn J, Anselm I, Bridgemohan C, Cox GF, et al. Microdeletion/duplication at 15q13.2q13.3 among individuals with features of autism and other neuropsychiatric disorders. J Med Genet. 2009;46(4):242-8.

Moreno-De-Luca D; SGENE Consortium, Mulle JG; Simons Simplex Collection Genetics Consortium, Kaminsky EB, Sanders SJ; et al. Deletion 17q12 is a recurrent copy number variant that confers high risk of autism and schizophrenia. Am J Hum Genet. 2010;87(5):618-30.

Mukaddes NM, Herguner S. Autistic disorder and 22q11.2 duplication. World J Biol Psychiatry. 2007;8(2):127-30.

Mulle JG, Dodd AF, McGrath JA, Wolyniec PS, Mitchell AA, Shetty AC, et al. Microdeletions of 3q29 confer high risk for schizophrenia. Am J Hum Genet. 2010;87(2):229-36.

Kieran C. Murphy, Peter J. Scambler (eds): Velo-cardio-facial syndrome: a model for understanding microdeletion disorders. Cambridge University Press, Cambridge, 2005.

Murthy SK, Nygren AO, El Shakankiry HM, Schouten JP, Al Khayat AI, Ridha A, et al. Detection of a novel familial deletion of four genes between BP1 and BP2 of the Prader-Willi/Angelman syndrome critical region by oligo-array CGH in a child with neurological disorder and speech impairment. Cytogenet Genome Res. 2007;116(1-2):135-40.

Nagamani SC, Erez A, Shen J, Li C, Roeder E, Cox S, Karaviti L, et al. Clinical spectrum associated with recurrent genomic rearrangements in chromosome 17q12. Eur J Hum Genet. 2010;18(3):278-84.

Niklasson L, Rasmussen P, Oskarsdóttir S, Gillberg C. Autism, ADHD, mental retardation and behavior problems in 100 individuals with 22q11 deletion syndrome. Res Dev Disabil. 2009;30(4):763-73.

Ogilvie CM, Moore J, Daker M, Palferman S, Docherty Z. Chromosome 22q11 deletions are not found in autistic patients identified using strict diagnostic criteria. IMGSAC. International Molecular Genetics Study of Autism Consortium. Am J Med Genet. 2000;96(1):15-7.

Ou Z, Berg JS, Yonath H, Enciso VB, Miller DT, Picker J, et al. Microduplications of 22q11.2 are frequently inherited and are associated with variable phenotypes. Genet Med. 2008;10(4):267-77.

Pinto D, Pagnamenta AT, Klei L, Anney R, Merico D, Regan R, et al. Functional impact of global rare copy number variation in autism spectrum disorders. Nature. 2010;466(7304):368-72.

Prasad SE, Howley S, Murphy KC. Candidate genes and the behavioral phenotype in 22q11.2 deletion syndrome. Dev Disabil Res Rev. 2008;14(1):26-34.

Ramalingam A, Zhou XG, Fiedler SD, Brawner SJ, Joyce JM, Liu HY, et al. 16p13.11 duplication is a risk factor for a wide spectrum of neuropsychiatric disorders. J Hum Genet. 2011;56(7):541-4.

Ramelli GP, Silacci C, Ferrarini A, Cattaneo C, Visconti P, Pescia G. Microduplication 22q11.2 in a child with autism spectrum disorder: clinical and genetic study. Dev Med Child Neurol. 2008;50(12):953-5.

Rosenfeld JA, Coppinger J, Bejjani BA, Girirajan S, Eichler EE, Shaffer LG, et al. Speech delays and behavioral problems are the predominant features in individuals with developmental delays and 16p11.2 microdeletions and microduplications. J Neurodev Disord. 2010;2(1):26-38.

Sanders SJ, Ercan-Sencicek AG, Hus V, Luo R, Murtha MT, Moreno-De-Luca D, et al. Multiple recurrent de novo CNVs, including duplications of the 7q11.23 Williams syndrome region, are strongly associated with autism. Neuron. 2011;70(5):863-85.

Schroer RJ, Phelan MC, Michaelis RC, Crawford EC, Skinner SA, Cuccaro M, et al. Autism and maternally derived aberrations of chromosome 15q.

Am J Med Genet. 1998;76(4):327-36.

Sebat J, Lakshmi B, Malhotra D, Troge J, Lese-Martin C, Walsh T, et al. Strong association of de novo copy number mutations with autism. Science. 2007;316(5823):445-9.

Shinawi M, Liu P, Kang SH, Shen J, Belmont JW, Scott DA, et al. Recurrent reciprocal 16p11.2 rearrangements associated with global developmental delay, behavioural problems, dysmorphism, epilepsy, and abnormal head size. J Med Genet. 2010;47(5):332-41.

Stefansson H, Rujescu D, Cichon S, Pietiläinen OP, Ingason A, Steinberg S, et al. Large recurrent microdeletions associated with schizophrenia. Nature. 2008;455(7210):232-6.

Autism Genome Project Consortium, Szatmari P, Paterson AD, Zwaigenbaum L, Roberts W, Brian J, et al. Mapping autism risk loci using genetic linkage and chromosomal rearrangements. Nat Genet. 2007;39(3):319-28.

Ullmann R, Turner G, Kirchhoff M, Chen W, Tonge B, Rosenberg C, et al. Array CGH identifies reciprocal 16p13.1 duplications and deletions that predispose to autism and/or mental retardation. Hum Mutat. 2007;28(7):674-82.

van Bon BW, Mefford HC, Menten B, Koolen DA, Sharp AJ, Nillesen WM, et al. Further delineation of the 15q13 microdeletion and duplication syndromes: a clinical spectrum varying from non-pathogenic to a severe outcome. J Med Genet. 2009;46(8):511-23.

Veenstra-VanderWeele J, Cook EH Jr. Molecular genetics of autism spectrum disorder. Mol Psychiatry. 2004;9(9):819-32.

Vorstman JA, Morcus ME, Duijff SN, Klaassen PW, Heineman-de Boer JA, Beemer FA, et al. The 22q11.2 deletion in children: high rate of autistic disorders and early onset of psychotic symptoms. J Am Acad Child Adolesc Psychiatry. 2006;45(9):1104-13.

von der Lippe C, Rustad C, Heimdal K, Rødningen OK. 15q11.2 microdeletion - seven new patients with delayed development and/or behavioural problems. Eur J Med Genet. 2011;54(3):357-60.

Weiss LA, Shen Y, Korn JM, Arking DE, Miller DT, Fossdal R, et al. Association between microdeletion and microduplication at 16p11.2 and autism. N Engl J Med. 2008;358(7):667-75.

Willatt L, Cox J, Barber J, Cabanas ED, Collins A, Donnai D, et al. 3q29 microdeletion syndrome: clinical and molecular characterization of a new syndrome. Am J Hum Genet. 2005;77(1):154-60.
